# Enzyme‐Responsive Nanoparachute for Targeted miRNA Delivery: A Protective Strategy Against Acute Liver and Kidney Injury

**DOI:** 10.1002/advs.202411210

**Published:** 2024-12-24

**Authors:** Songhang Li, Yuxuan Zhao, Xiaoying Lyu, Ye Chen, Tao Zhang, Shiyu Lin, Zhiqiang Liu, Xiaoxiao Cai, Taoran Tian, Yunfeng Lin

**Affiliations:** ^1^ State Key Laboratory of Oral Diseases National Clinical Research Center for Oral Diseases West China Hospital of Stomatology Sichuan University Chengdu 610041 P. R. China; ^2^ Department of Oral Surgery Shanghai Ninth People's Hospital Shanghai Jiao Tong University School of Medicine College of Stomatology Shanghai Jiao Tong University Shanghai 200011 P. R. China; ^3^ College of Biomedical Engineering Sichuan University Chengdu 610041 P. R. China

**Keywords:** acute kidney injury, acute liver failure, enzyme‐responsive, MiRNA‐125, nanoparachute, tetrahedral frame nucleic acid

## Abstract

MicroRNA (miRNA)‐based therapy holds significant potential; however, its structural limitations pose a challenge to the full exploitation of its biomedical functionality. Framework nucleic acids are promising owing to their transportability, biocompatibility, and functional editability. MiRNA‐125 is embedded into a nucleic acid framework to create an enzyme‐responsive nanoparachute (NP), enhancing the miRNA loading capacity while preserving the attributes of small‐scale framework nucleic acids and circumventing the uncertainty related to RNA exposure in conventional loading methods. An enzyme‐sensitive sequence is designed in NP as a bioswitchable apparatus for cargo miRNAs release. NP is compared with conventional delivery modes and delivery vehicles, confirming its excellent transportability and sustained release properties. Moreover, NP confers good enzyme and serum resistance to the cargo miRNAs. Simultaneously, it can easily deliver miRNA‐125 to liver and kidney lesions owing to its passive targeting properties. This allows for Keap1/Nrf2 pathway regulation and p53 protein targeting in the affected tissues. Additionally, NP negatively regulates the expression of Bax and Caspase‐3. These combined actions help to inhibit oxidation, prevent cell cycle arrest, and reduce the apoptosis of liver and kidney cells. Consequently, this strategy offers a potential treatment for acute liver and kidney injury.

## Graphical abstract

Framework nucleic acids are promising owing to their transportability, biocompatibility, and functional editability. MiRNA‐125 are embedded into a nucleic acid framework to create an enzyme‐responsive nanoparachute. By utilizing the passive targeting property of NP, miRNA‐125 can be easily delivered to liver and kidney lesions. This strategy offers a potential treatment for acute liver and kidney injury.

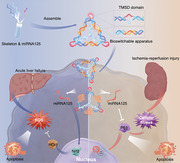

## 1 Introduction

Liver and kidney function failures can be attributed to a multitude of factors and can lead to profound damage and extensive cellular necrosis.^[^ ^1^ ^]^ Consequently, synthesis, secretion, conversion, and detoxification in the liver may be impaired, culminating in renal dysfunction or systemic decompensation.^[^ ^2^ ^]^ The role of microRNAs (miRNAs) in various physiological and pathological processes, including embryonic development, apoptosis, and disease, underscores their importance as nucleic acid drugs.^[^ ^3^ ^]^ MiRNAs exhibit a wide range of functions and exert diverse biological effects on different tissues through direct or indirect regulation.^[^ ^4^ ^]^ For example, miRNA‐125 enhances the expression of nuclear factor‐E2‐related factor 2 (Nrf‐2), a known regulatory factor of acute liver failure (ALF), by directly modulating Kelch‐like ECH‐associated protein 1 (Keap1) in the liver, thereby ameliorating ALF.^[^ ^5^ ^]^ In acute kidney injury (AKI), miRNA‐125 can directly suppress p53 protein expression, leading to CDK1 and Cyclin B1 upregulation to rescue G2/M phase arrest and Bcl‐2 and Bax modulation to inhibit apoptosis.^[^ ^6^ ^]^

However, the current development of miRNA‐based nucleic acid drugs often overlooks crucial aspects of miRNA functional diversity.^[^ ^2^, ^4^, ^7^ ^]^ In addition, the inherent structural limitations of miRNAs render them susceptible to degradation by ribonucleases present in the bloodstream when used in vivo, hindering the widespread application of miRNA therapy.^[^ ^8^ ^]^ Although methods such as viral packaging, liposomes, and dendrimers have been explored for miRNA delivery, concerns regarding biosafety and uncertain delivery efficiency have prevented most therapies from progressing beyond the preclinical stage.^[^ ^9^ ^]^ Despite significant progress over the past decade in mitigating nuclease resistance associated with miRNA drugs, an urgent need to develop efficient in vivo delivery vectors remains. Most chemically modified miRNA drugs demonstrate restricted tissue distribution due to rapid metabolism by the hepatic and renal systems, followed by urinary excretion.^[^ ^10^ ^]^ This limitation often necessitates higher doses to achieve therapeutic objectives, thereby increasing the risks of off‐target effects. Consequently, addressing the challenge of in vivo delivery is crucial to achieving the desired therapeutic efficacy of miRNAs.

DNA nanotechnology offers unparalleled control over the size and geometry of DNA nanostructures through molecular recognition via the Watson–Crick base complementarity principle.^[^ ^11^ ^]^ Tetrahedral frame nucleic acid (tFNA), the simplest form of the DNA nanostructure, exhibits low immunogenicity, excellent biocompatibility, and remarkable stability, making it highly suitable for in vivo applications.^[^ ^12^ ^]^ Moreover, tFNA is easily prepared and highly productive, making it a promising solution for the in vivo delivery of miRNAs.^[^ ^13^ ^]^ Passive targeting of the liver and kidney by tFNA leads to the accumulation of miRNAs in these organs. This suggests that tFNA could improve the efficacy of miRNA therapy by directly delivering therapeutic miRNAs to these sites, thereby enhancing treatment outcomes and reducing off‐target effects.^[^ ^14^ ^]^ Additionally, tFNA has anti‐inflammatory and antioxidant properties, enhancing its effectiveness as a carrier for miRNAs. These compelling biological activities highlight the multifunctionality of tFNA as a carrier of miRNAs associated with liver and kidney injury. Previous research has shown that tFNA can modulate the p53 pathway to alleviate acute liver injury and can act as an effective reactive oxygen species (ROS) scavenger for treating AKI.^[^ ^14a^ ^]^ These findings, combined with the therapeutic potential of miRNA‐125, highlight the promise of using tFNA in synergistic treatments for ALF and AKI.

**Figure 1:**
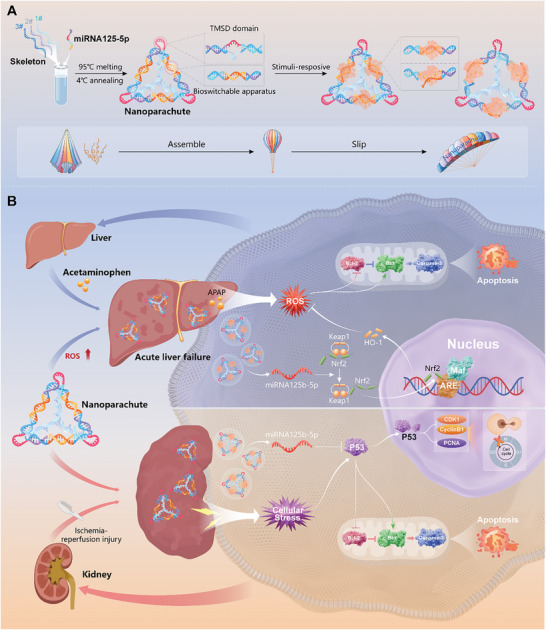
Schematic illustration of the assembly and application of the nanoparachute (NP). A) NP synthesized using a one‐pot method opening its structures in response to stimuli, similar to the assembly and deployment of a parachute, ultimately becoming a protective agent for acute liver and kidney injury. B) NP inhibited oxidative stress, G2/M phase arrest, and apoptosis, exhibiting therapeutic effects against ALF and AKI.

However, in the past, the transport of miRNAs, siRNAs, or aptamers using tFNA often involved loading the cargo onto the apex or hanging it from the sidearm of the tFNA.^[^ ^13^, ^15^ ^]^ This approach not only increased the size of the nanostructure but also exposed the nucleic acid drugs to substantial amounts of nucleic acid enzymes in circulation, introducing great uncertainty to the cargo transportation. Moreover, this static delivery mode hampers the controlled release of the miRNAs, and multiple factors significantly affect the efficiency of miRNA delivery.^[^ ^16^ ^]^ Here, we integrated miRNA‐125, which has diverse functionality, into the tFNA framework to ensure optimal protection of the fragile miRNAs. Drawing inspiration from parachutes, we incorporated a bioswitchable apparatus at the termini of each miRNA‐125 molecule; these enzyme‐responsive switches are activated by RNase‐H upon delivery of the nanostructure to its intended destination.^[^ ^17^ ^]^ This mechanism facilitates the unfurling of the parachute and enhances recognition between miRNA‐125 and its target molecules.^[^ ^18^ ^]^ We refer to this innovative structure as the nanoparachute (NP) (**Figure** **1**). The unique tetrahedral topology of the parachute enables efficient miRNA transportation and the passive targeting of liver and kidney tissues. In this study, we demonstrated that NP can passively target the livers and kidneys for efficient transportation and preferential accumulation in these organs. Compared with traditional in vivo delivery vectors, NP significantly prolongs the retention time of miRNAs in the body. Moreover, our delivery platform technology exhibited remarkable in vivo therapeutic efficacy in ALF and AKI models (Figure 1). We successfully developed a highly efficient, stable, and biocompatible miRNA delivery platform that supports the enhancement of miRNA delivery technology and innovative treatments for liver‐ and kidney‐related diseases.

## 2 Results and Discussion

### 2.1 Design, Fabrication, and Characterization of NP

**Figure 2:**
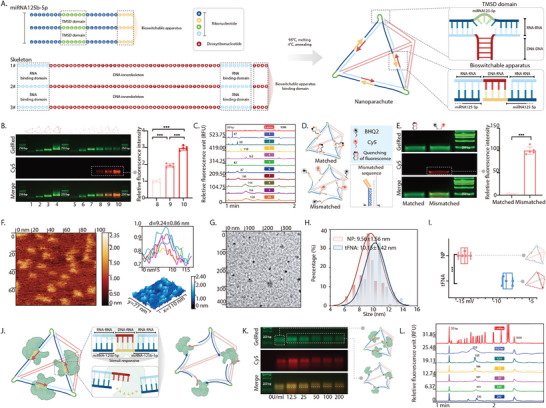
Design, fabrication, and characterization of NP. A) Schematic display of the fabrication of NP. B) AGE showing the stepwise fabrication of NP and tFNA. MiRNA‐125 was successfully loaded on NP, as indicated by the enhanced Cy5 fluorescence (lane 1: tFNA‐S1; lane 2: tFNA‐S1+tFNA‐S2; lane 3: tFNA‐S1+tFNA‐S2+tFNA‐S3; lane 4: tFNA‐S1+tFNA‐S2+tFNA‐S3+tFNA‐S4; lane 5: S1; lane 6: S1+S2; lane 7: S1+S2+S3; lane 8: S1+S2+S3+Cy5‐miRNA‐125; lane 9: S1+S2+S3+2×Cy5‐miRNA‐125; lane 10: S1+S2+S3+3×Cy5‐miRNA‐125). C) CGE showing the stepwise fabrication of NP and tFNA. D) Schematic illustration of the matched‐ and mismatched‐NP. E) In AGE, the matched‐NP exhibited minimal Cy5 fluorescence, suggesting the successful integration of the structure. F) AFM image showing the small size of NP. G) TEM image showing the small‐sized NP distributed evenly. H) Hydrodynamic size analysis showing the similar sizes of NP and tFNA. I) The zeta potential of NP showing a negative electric charge. J) Schematic illustration of NP opening in response to stimuli. K) AGE showing NP undergoing a conformational change in response to stimuli. L) CGE analysis validated the observed structural shifts observed in the AGE. Data were statistically evaluated using a one‐tailed Student's *t*‐test between two groups or a one‐way ANOVA and post‐hoc analysis (Sidak. test) for multiple comparisons. Data are presented as the mean ± standard deviation (SD) (*n* ≥ 3). ***p < 0.001.

To ensure the success of the sequence design, we developed a structural nanoparticle design based on a tFNA that was 21 base pairs long. We replaced one of the four DNA strands with three miRNA‐125 sequences and extended the 3′ end of each miRNA‐125 sequence by four nucleotides to form part of the bioswitchable apparatus. The complementary segments of the remaining three DNA skeleton strands that paired with the miRNAs were replaced with ribonucleotides, forming the RNA‐binding domain (**Figure** **2**A). Meanwhile, the complementary segments that interacted with the bioswitchable apparatus remained as deoxyribonucleotides. This design ensured that the bioswitchable apparatus could be activated by RNase H, which is prevalent in mammalian cells, ultimately leading to the release of miRNAs from NP.^[^ ^17^ ^]^ At each apex where miRNA‐125 was involved in the formation, we designed five nucleotide bases that did not participate in base pairing, allowing miRNA‐125 to engage in a toehold‐mediated strand displacement reaction with its target (Figure 2A).^[^ ^18^ ^]^

Through this folding design, we created an NP that could embed miRNA‐125 within its tetrahedral topology, providing adequate protection. To intuitively observe the nanostructure more directly, we designed the 5′ end of each miRNA‐125 with Cy5. After a one‐pot annealing process, the assembled NP exhibited a length similar to that of tFNA (≈21 base pairs) as observed via agarose gel electrophoresis (AGE). The gradual enhancement of Cy5 fluorescence also indicated that miRNA‐125 was gradually loaded onto NP (the relative fluorescence intensity increased 1.00 ± 0.08‐fold, 1.94 ± 0.11‐fold, and 2.97 ± 0.14‐fold) (Figure 2B). NP also showed similar relative fluorescence values to tFNA in capillary gel electrophoresis (CGE), suggesting that they had similar structures (Figure 2C; Figure S1, Supporting Information). To verify that the structure of NP was consistent with our assumptions, we used Black Hole Quencher‐2 (BHQ‐2) to modify the 3′ ends of miRNA‐125 and mismatched miRNA‐125. We then synthesized matched‐ and mismatched‐NP using a one‐pot annealing process (Figure 2D). The successful synthesis and complete closure of the NP framework resulted in a Cy5 fluorescence intensity in the matched‐NP that was only 3.43 ± 0.99% of that in the mismatched‐NP (Figure 2E). Atomic force microscopy (AFM) and transmission electron microscopy (TEM) were used to observe the morphology of the nanoparticles (Figure 2F,G); NP was small and evenly distributed.

Subsequently, we used dynamic light scattering to precisely quantify the hydrodynamic size of the nanoparticles. The results revealed that, after embedding miRNA‐125 in the tetrahedral topology, the particle size was similar to that of the tFNA with a length of 21 base pairs (NP 9.56 ± 1.56 nm; tFNA 10.15 ± 1.42 nm) (Figure 2H). As the RNA structure replaces the original DNA framework in NP, it has a stronger negative charge than the tFNA (Figure 2I). To facilitate the binding of the NP‐loaded miRNA‐125 to the intracellular target, the bioswitchable apparatus in NP can be triggered by RNase H, a ubiquitous enzyme in mammalian cells, which degrades the RNA portion of the DNA–RNA hybrid chain in the bioswitchable apparatus, thereby releasing miRNAs from NP (Figure 2J). We then simulated the opening of the nanostructure in an extracellular environment and found that, as the enzymatic activity unit of RNase H in the incubation environment increased, the migration rate of the nanostructure gradually slowed. This confirmed that the 3D conformation of NP had changed (Figure 2K). CGE confirmed this result, indicating that the nanostructure entered the cell at a relatively small size and was then opened by RNase H to facilitate its binding to the intracellular target (Figure 2L; Figure S2, Supporting Information).

### 2.2 Metabolism and Distribution of NP in vivo

**Figure 3:**
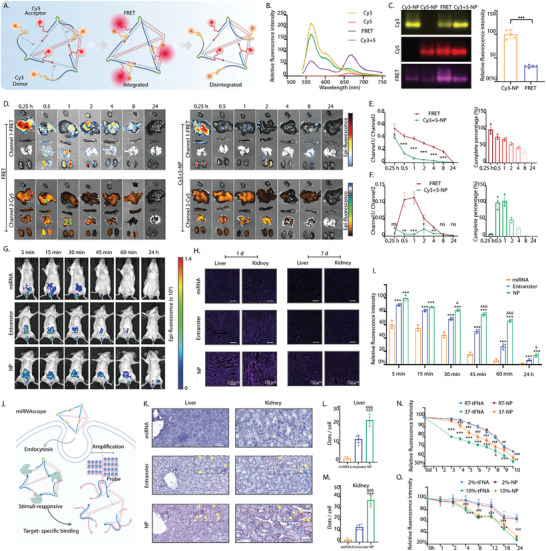
Distribution of NP and its advantages as a miRNA delivery vehicle. A) Design of Cy5 (acceptor) and Cy3 (donor) in NP. B) Relative fluorescence intensity showing the FRET results. C) AGE also confirming the FRET results. ***p < 0.001. D) Changes in the relative fluorescence intensity (Channel1‐FRET/Channel2‐Cy5) showing the metabolic status of NP in vivo. E,F) Changes in the relative fluorescence intensity (Channel1‐FRET/Channel2‐Cy5) in liver and kidney. *p < 0.05; **p < 0.01; ***p < 0.001. G) In vivo fluorescence signal changes were used to evaluate the metabolism of miRNA, Entranster, and NP. H) Fluorescence imaging of histological sections after injection of the miRNA, Entranster, and NP (Cy5: red; nucleus: blue) (Scale bar: 250 µm). I) Statistical analysis of relative fluorescence intensity. ***p < 0.001 compared with miRNA; *^&^p* < 0.05, *^&&&^p* < 0.001 compared with Entranster. J) Schematic diagram of miRNAscope. K) In both liver and kidney tissue slices, the NP group can be observed to have a large number of red dots, while the Entranster group also accumulated some red dots, but almost no red dots can be observed in the miRNA group (Scale bar: 50 µm). L,M) Statistical analysis of red dots per cell in liver and kidney. ***p < 0.001 compared with miRNA; *^&&&^p* < 0.001 compared with Entranster. N) Storage stability test showing similar stabilities of NP and tFNA at room temperature, but NP showed more outstanding stability than tFNA at 37 °C. O) Serum stability test showing the similar stability of NP and tFNA in both 2% and 10% serum concentrations. Data were statistically evaluated using a one‐tailed Student's *t*‐test between two groups or a one‐way ANOVA and *post hoc* analysis (Sidak. test) for multiple comparisons. Data are presented as the mean ± SD (*n* ≥ 3).

To clearly observe the metabolism and distribution of NP in vivo, the 5′ and 3′ ends of miRNA‐125 were modified with Cy5 (acceptor) and Cy3 (donor) (**Figure** **3**A). When NP is intact, Forster resonance energy transfer (FRET) occurs between the donor (Cy3) and acceptor (Cy5), leading to increased fluorescence at the acceptor (Cy5) and decreased fluorescence at the donor (Cy3) (Figure 3A). Upon disintegration of NP, FRET is disrupted, and the fluorescence of the acceptor (Cy5) is not enhanced. The changes in the fluorescence intensity of the acceptor/donor allowed us to clearly observe the metabolism and distribution of NP in vivo. We separately prepared Cy3‐NP, Cy5‐NP, and FRET‐NP and then mixed Cy3‐NP and Cy5‐NP in equal proportions to prepare Cy3+5‐NP. We measured the relative fluorescence intensities of the four samples and found that FRET‐NP exhibited lower fluorescence at 570 nm and higher fluorescence at 670 nm compared with the other samples. This shift in fluorescence confirmed that FRET occurred between the Cy3 donor and Cy5 acceptor in FRET‐NP (Figure 3B). These results were validated by AGE (Figure 3C). Subsequently, FRET‐NP and Cy3+5‐NP were injected into mice in vivo, and the metabolic status of NP in vivo was observed by monitoring the changes in the relative fluorescence intensity (Channel1‐FRET/Channel2‐Cy5) (Figure 3D). At 8 h after injection, the proportion of intact NP in the liver gradually decreased; in the kidneys, it reached its peak at 0.5–1 h and then gradually decreased. At 24 h, no integrated nanoparticles were detected in either the liver or kidneys (Figure 3E,F).

### 2.3 Advantages of NP as a miRNA Delivery Vehicle

To further demonstrate the significant potential of NP in delivering miRNA, we compared it with another in vivo RNA transfection reagent, Entranster. Cy5‐modified miRNA‐125 was used to prepare the miRNA, Entranster, and NP groups, and an in vivo imaging system was used at six‐time points after injection (Figure 3G). Direct injection of naked miRNA‐125 exhibited almost no fluorescence within 45 min. The Entranster and NP groups showed similar relative fluorescence intensities within 15 min, but the relative fluorescence intensity of the NP group was significantly higher than that of the Entranster group at 30 min (Figure 3I). When observing the fluorescence of the liver and kidney slices, the Cy5 fluorescence in the NP group was significantly stronger than that in the Entranster group, whereas the fluorescence signal in the miRNA group was hardly detectable (Figure 3H; Figure S3, Supporting Information). MiRNAscope is a stable RNA in situ hybridization assay that detects the spatial distribution of miRNA‐125 in tissues via highly specific binding of probes to miRNA‐125 (Figure 3J). In both the liver and kidney tissue slices, the NP group exhibited a large number of red dots, the Entranster group also showed some red dots, but almost none were observed in the miRNA group (Figure 3K). Analysis of the number of red dots in each cell revealed statistical differences between the NP and Entranster groups in both the liver and kidney samples. These results also confirm that NP exhibited superior miRNA delivery efficiency compared with the commercially available RNA drug delivery vehicle Entranster (Figure 3L,M).

The miRNAs within a miRNA delivery vehicle must be protected; therefore, we evaluated the stability of NP. In the storage stability test, NP and tFNA showed similar stability at room temperature, but NP showed outstanding stability at 37 °C, which may be related to the RNA double‐stranded structure present in its internal composition (Figure 3N). In the serum stability test, NP showed similar stability results to tFNA at both 2% and 10% serum concentrations (Figure 3O). The advantages of NP in efficiently delivering miRNAs undoubtedly advance the prospects for miRNA‐related drugs limited by unstable miRNAs.

### 2.4 Therapeutic Effect of NP on APAP‐Induced ALF

**Figure 4:**
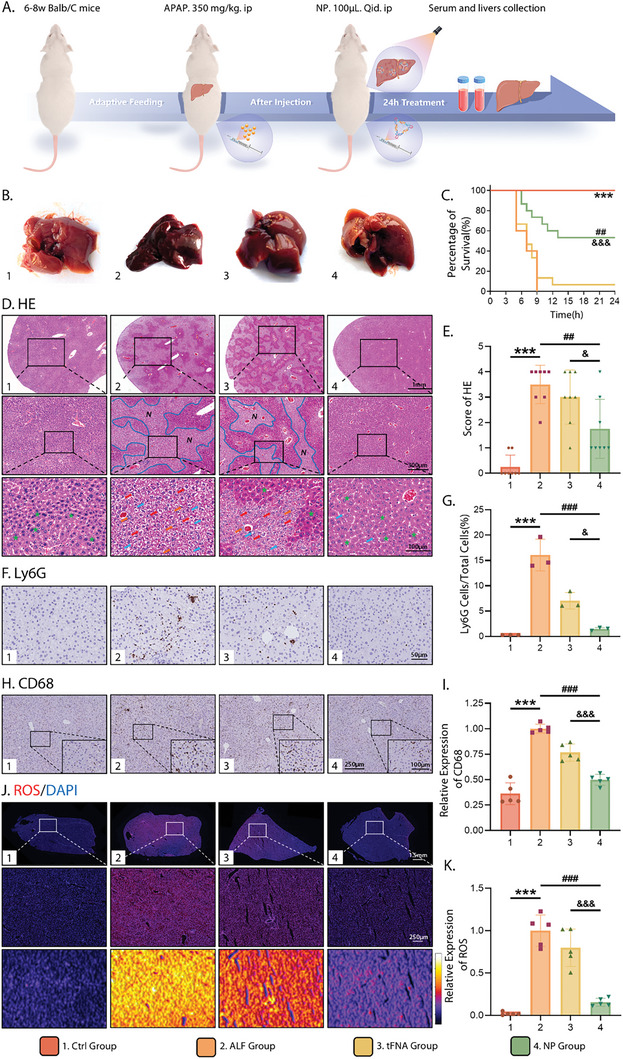
Therapeutical effect of NP on APAP‐induced ALF in mice. A) Methods and time nodes for drug treatment of ALF in vivo. B) Gross morphology of livers from each group. C) Kaplan–Meier survival analyses of mice after different treatments. D) Images of livers with HE staining (Scale bar: 1 mm, 300 µm, and 100 µm). Green asterisks: normal hepatocytes; red arrows: vacuolar degeneration of hepatocytes; blue arrows: lipid droplets, suggestive of hepatocyte steatosis; orange arrows: necrotic hepatocytes; N: necrotic area. E) Histopathological scoring of liver sections based on HE staining. F,H) Representative images of Ly6G and CD68 expression in livers based on immunohistochemistry staining (Scale bars: 50, 100, and 250 µm). G) Quantitative analysis of the percentage of Ly6G‐positive cells. I) Quantitative analysis of CD68 expression levels. J) Immunofluorescence images of livers with DHE staining (ROS: red; nucleus: blue; 3D thermal imaging: reconstruction of fluorescence intensity of ROS) (Scale bars: 1.5 mm and 250 µm). K) Quantitative analysis of the relative fluorescence intensity of ROS. All data were performed using one‐way ANOVA and *post hoc* analysis (Sidak. test) for multiple comparisons and presented as mean ± SD (*n* ≥ 3). The error bars represent the SD. Statistical analysis: (*) compared with the control group; *p < 0.05, **p < 0.01, ***p < 0.001; (^#^) compared with the AKI group; ^#^ *p* < 0.05, ^##^ *p* < 0.01, ^###^ *p* < 0.001; (^&^) compared with the tFNA group; ^&^ *p* < 0.05, ^&&^ *p* < 0.01, ^&&&^ *p* < 0.001.

Given the superior stability and targeting ability of NP, we evaluated its in vivo therapeutic potential. As outlined in **Figure** **4**A, we utilized a well‐established ALF mouse model based on the excessive application of acetaminophen (N‐acetyl‐p‐aminophenol, APAP),^[^ ^5^, ^19^ ^]^ a dose‐dependent hepatotoxicant commonly associated with drug‐related ALF.^[^ ^20^ ^]^ Tissue samples were collected after 24 h. The mortalities in different groups of mice are shown in Figure 4C, and the Kaplan–Meier survival curve analyses showed a 0% 24 h survival rate in the ALF group, which substantially increased to ≈50% following NP administration, indicating a distinct survival benefit for mice undergoing NP treatment. Supporting this, we photographed the gross morphology of the livers in each group (Figure 4B). The liver surface of the control group was smooth and glossy, with a uniform texture and homogenous bright ruddy color. However, the livers of APAP‐treated mice displayed an uneven, rough, and shrunken appearance with a hardened texture and darkened color, indicating successful establishment of the ALF model. Both tFNA and NP treatments alleviated the pathological state of the livers caused by APAP, whereas the livers in the NP group appeared visibly healthier, indicating that NP effectively prevented APAP‐induced liver injury, thereby increasing mouse survival rates.

HE staining of the liver tissues in Figure 4D was subsequently performed to visually assess the APAP‐induced hepatotoxicity, as well as the therapeutic performance of NP on ALF. Histologically, severe hepatic damage was observed in the livers of the ALF group, with disrupted liver architecture and large patches of necrotic regions with massive vacuolar degeneration and hepatocyte steatosis. The necrosis of the liver decreased after tFNA intervention but was still serious, whereas little damage was observed in the NP group, which displayed healthy histopathology accompanied by small amounts of lipid droplets. Histopathological scoring of liver sections according to the severity of liver damage further revealed that treatment with NP indeed contributed to alleviating APAP‐induced liver failure (Figure 4E). Necro‐inflammation has been regarded as a common indicator of ALF.^[^ ^21^ ^]^ To evaluate the infiltration of inflammatory cells into tissues linked to liver injury, we monitored the infiltration of neutrophils by Ly6G staining and the expression of the macrophage surface‐specific marker, CD68, by immunohistochemistry. As exhibited in Figure 4F,H, the Ly6G‐positive and CD68‐positive cells (dark brown‐stained) represented infiltrating neutrophils and macrophages in the liver tissue, respectively. The livers of the ALF group displayed enhanced severe neutrophil infiltration and upregulated CD68 expression compared to those of the control group, suggestive of the aggravation of necro‐inflammation. Treatment with tFNA and NP ameliorated liver inflammation, and the therapeutic effect of NP was more evident, with a remarkable decrease in the percentage of Ly6G‐positive cells and CD68 expression levels. Statistical analyses of the histological results further verified these conclusions (Figure 4G,I).

Furthermore, recent experimental evidence suggests that the accumulation of ROS is a critical pathological characteristic closely associated with early‐stage APAP‐induced ALF.^[^ ^22^ ^]^ Excessive ROS production can lead to DNA and mitochondrial destruction, rapid inflammation development, massive ALT release, and eventually to hepatocyte necrosis.^[^ ^23^ ^]^ Therefore, blocking ROS production has been demonstrated as a potential treatment for early ALF. To determine whether NP contributes to scavenging APAP‐induced ROS, we performed dihydroethidium (DHE) staining of liver tissues. As expected, the level of ROS, as indicated by red fluorescence, sharply increased after APAP injection, followed by a significant decrease with NP treatment. This confirmed the remarkable protective capacity of NP against oxidative damage in vivo (Figure 4J,K). In summary, the results from serological and histological analyses demonstrate that NP effectively suppresses hepatic necrosis, reduces inflammation, and maintains ROS homeostasis, establishing its strong therapeutic potential for ALF.

### 2.5 NP Inhibits APAP‐Induced Oxidation by Regulating the Keap1/Nrf2 Pathway

**Figure 5:**
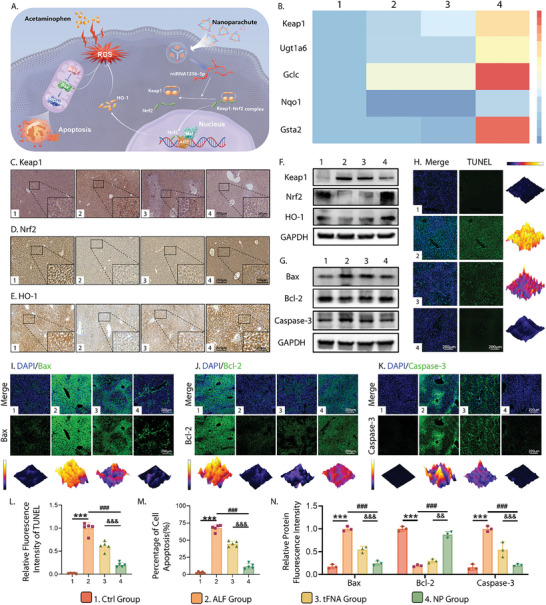
Inhibitory effect of NP on APAP‐induced oxidative stress and apoptosis of hepatocytes. A) Schematic diagram of mechanisms involved in the protective effect of NP on APAP‐intoxicated hepatocytes. B) RT‐qPCR analysis of the expression of Keap1 and its related downstream genes. C–E) Representative images of Keap1, Nrf2, and HO‐1 expression in livers based on immunohistochemistry staining (Scale bars: 50, 100, and 200 µm). F) Protein expression in western blotting analysis of Keap1, Nrf2, and HO‐1. G) Protein expression in western blotting analysis of apoptosis‐related proteins: Bax, Bcl‐2, and Caspase‐3. H) Immunofluorescence images of livers with TUNEL staining (TUNEL: green; nucleus: blue; 3D thermal imaging: reconstruction of fluorescence intensity of TUNEL) (Scale bar: 200 µm). I–K) Immunofluorescence images of Bax, Bcl‐2, and Caspase‐3 expression (Bax, Bcl‐2, and Caspase‐3: green; nucleus: blue; 3D thermal imaging: reconstruction of fluorescence intensity of Bax, Bcl‐2, and Caspase‐3) (Scale bar: 200 µm). L) Quantitative analysis of relative TUNEL fluorescence intensity based on TUNEL staining. M) Quantitative analysis of percentage of TUNEL‐positive cells among the total cells based on TUNEL staining. N) Quantitative analysis of relative fluorescence intensity of Bax, Bcl‐2, and Caspase‐3. All data were tested using one‐way ANOVA and *post hoc* analysis (Sidak. test) for multiple comparisons and presented as the mean ± standard deviation (SD) (*n* ≥ 3). Error bars represent the SD. Statistical analysis: (*) compared with the control group; *p < 0.05, **p < 0.01, ***p < 0.001 (^#^) compared with the AKI group; ^#^ *p* < 0.05, ^##^ *p* < 0.01, ^###^ *p* < 0.001 (^&^) compared with the tFNA group; ^&^ *p* < 0.05, ^&&^ *p* < 0.01, ^&&&^ *p* < 0.001.

Previous studies have shown that APAP overdose triggers ROS‐mediated oxidative injury in hepatocytes.^[^ ^24^ ^]^ As depicted in **Figure** **5**A, the Keap1/nuclear factor erythroid 2‐related factor 2 (Nrf2) signaling pathway, a self‐protection mechanism, is a critical positive feedback regulatory pathway for systemic inflammation, with the functions of significantly blocking endogenous or exogenous oxidative stress and maintaining intracellular redox homeostasis.^[^ ^25^ ^]^ During this process, the expression of heme oxygenase‐1 (HO‐1), which is recognized as a pivotal protective antioxidant involved in pro‐inflammatory factor suppression and ROS elimination, is activated. Therefore, the Keap1/Nrf2 signaling pathway has become a critical therapeutic target for diverse oxidative stress‐related diseases, especially ALF.^[^ ^26^ ^]^ Based on the accumulated achievements of long‐term research at the genetic level in recent decades, RNA therapy has emerged rapidly and revealed remarkable application potential with high target specificity, pharmacokinetic and pharmacodynamic predictability, and excellent biosafety, making it a powerful alternative strategy for treating diseases beyond the reach of conventional medicines.^[^ ^27^ ^]^ Previous studies have demonstrated that miRNA‐125 is an effective regulator of the Keap1/Nrf2 signaling pathway, which can target the Keap1 protein and promote the dissociation of Keap1 and Nrf2, followed by the activation of its downstream anti‐ROS pathway, ultimately achieving therapeutic performance against APAP‐induced ALF.^[^ ^5^, ^28^ ^]^ Therefore, the regulation of the Keap1/Nrf2 pathway by increasing the expression of intracellular miRNA‐125 is a potential alternative therapy for ALF.

To investigate the mechanism of NP in treating APAP‐induced ALF mice, we assessed the contribution of the Keap1/Nrf2 axis using real‐time quantitative polymerase chain reaction (RT‐qPCR), western blotting, and immunohistochemical (IHC) staining. PCR analysis (Figure 5B) revealed that NP upregulated anti‐inflammatory and antioxidant genes, such as Ugt1a6, Gclc, Nqo1, and Gsta2,^[^ ^29^ ^]^ downstream of the Keap1/Nrf2 pathway, indicating activation of this pathway. Western blotting (Figure 5F; Figure S4A, Supporting Information) revealed that ALF increased Keap1, decreased Nrf2 and HO‐1, and rapidly depleted ROS in ALF mice. NP treatment reversed these effects by inhibiting Keap1 and restoring the Nrf2 and HO‐1 levels, aiding in ROS elimination. The IHC staining results in Figure 5C–E, and Figure S4B (Supporting Information) further confirmed these findings, showing increased Keap1 expression in the ALF group, which was reduced by NP treatment. The Nrf2 and HO‐1 levels were the highest in the NP group, consistent with the western blotting results. These findings indicate that the therapeutic effect of NP on ALF is due to the activation of the Keap1/Nrf2 signaling pathway.

### 2.6 NP Inhibits Hepatocyte Apoptosis Induced by APAP

Oxidative stress and the resulting extensive hepatocellular apoptosis are the main pathological mechanisms involved in the progression of ALF; they complement each other and jointly mediate hepatocyte necrosis.^[^ ^15^, ^30^ ^]^ As presented in Figure 4A, the accumulation of ROS in hepatocytes induced by excessive APAP is a substantial contributor to mitochondria‐mediated cell apoptosis. The superfluous ROS facilitate the production of the pro‐apoptotic proteins Bax and Caspase‐3 and block transcription of the anti‐apoptotic protein Bcl‐2. Hence, transferase dUTP nick‐end labeling (TUNEL) staining was employed to evaluate the anti‐apoptotic properties of NP (Figure 5H). Massive DNA strand breaks (corresponding to apoptotic cells) were observed in the hepatocytes of the ALF group, which were labeled with fluorescein‐dUTP under the catalysis of terminal deoxynucleotide transferase, emitting green fluorescence. TFNA decreased the production of green fluorescence to a certain extent, but the effect was much weaker than that of NP. Combined with the quantitative analyses of the TUNEL fluorescence results in Figure 5L,M, it was confirmed that NP therapy could significantly alleviate the apoptosis of injured hepatocytes, as evidenced by the notable decline in green fluorescence intensity and proportion of apoptotic cells.

The potent anti‐apoptotic activities of NP may be attributed both to its excellent ROS‐scavenging properties and the beneficial anti‐apoptotic effects of miRNA‐125. We examined its role in the mitochondrial apoptotic pathway using western blotting to explore the expression levels of apoptosis‐related proteins in each group (Figure 5G; Figure S4C, Supporting Information). As expected, NP treatment effectively reversed the upregulation of Bax and Caspase‐3 in APAP‐intoxicated hepatocytes and suppressed the downregulation of Bcl‐2, which was beneficial for alleviating apoptosis. Immunofluorescent staining of these proteins confirmed these results (Figure 5I–K,N). Bax and Caspase‐3 exhibited the brightest green fluorescence in the ALF group and were significantly weakened after treatment with tFNA and NP; however, the degree of attenuation in the tFNA group was significantly lower than that in the NP group. The opposite was observed for Bcl‐2 expression. In summary, the inhibitory effect of NP on the ALF resulted from the synergistic suppression of intracellular oxidative stress and apoptosis. NP exerts highly potent ROS‐scavenging activities by activating the Keap1/Nrf2 signaling pathway and blocking hepatocyte apoptosis by regulating the mitochondrial apoptosis pathway.

### 2.7 Therapeutic Effect of NP on I/R Induced AKI

**Figure 6:**
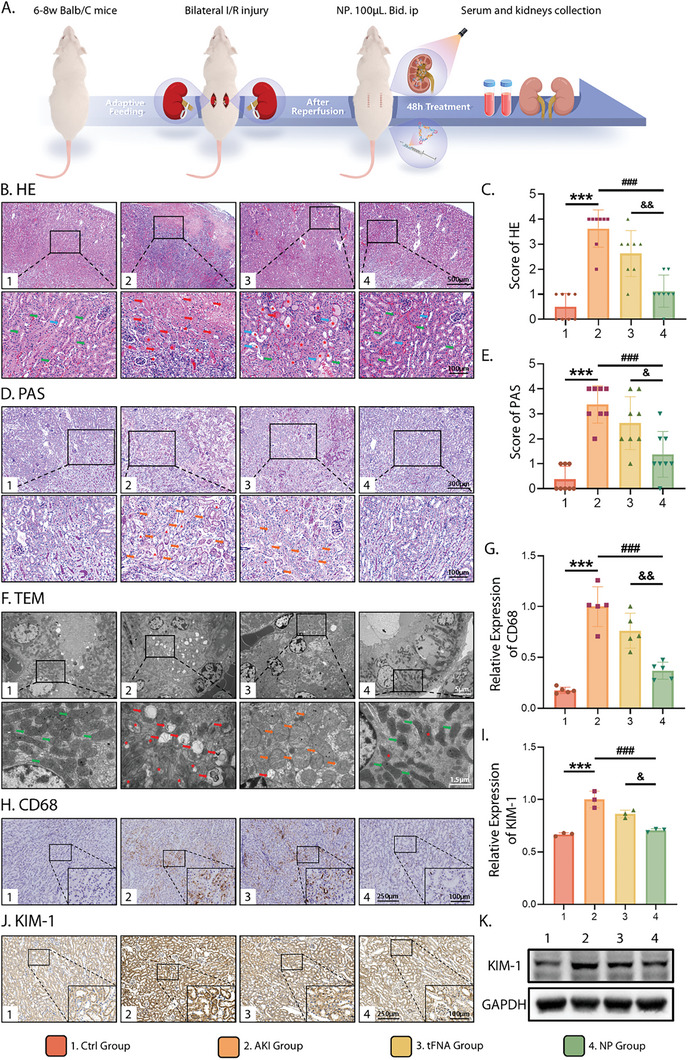
Therapeutical effect of NP on APAP‐induced ALF in mice. A) Schematic display of methods and time nodes for drug treatment of AKI in vivo. B,D) Images of kidneys with HE and PAS staining (Scale bar: 100, 300, and 500 µm). Green arrows: healthy renal tubules; red arrows: coagulated necrosis of renal tubules; red asterisks: hyaline casts; red triangle: mucous exudate; blue arrows: renal tubule dilatation; orange arrows: loss of brush borders. C) Histopathological scoring of renal sections based on HE staining. E) Histopathological scoring of kidney sections based on PAS staining. F) Representative TEM image of mitochondria in kidneys from different groups (Scale bars: 5 and 1.5 µm). Green arrows: healthy mitochondria; red arrows: fragmentation of mitochondria with matrix substance extravasation; red asterisks: disorganization or loss of mitochondrial cristae; orange arrows: mitochondrial swelling. G) Quantitative analysis of CD68 expression based on immunohistochemistry results. H) Immunohistochemistry images of CD68 expression (Scale bars: 250 and 100 µm). I) Quantitative analysis of KIM‐1 expression based on immunohistochemistry results. J) Immunohistochemistry images of KIM‐1 expression (Scale bars: 250 and 100 µm). L) Protein expression of western blotting analysis of KIM‐1. All data were tested using one‐way ANOVA and *post hoc* analysis (Sidak. test) for multiple comparisons and presented as the mean ± standard deviation (SD) (*n* ≥ 3). The error bars represent the SD. Statistical analysis: (*) compared with the control group; *p < 0.05, **p < 0.01, ***p < 0.001; (^#^) compared with the AKI group; ^#^ *p* < 0.05, ^##^ *p* < 0.01, ^###^ *p* < 0.001; (^&^) compared with the tFNA group; ^&^ *p* < 0.05, ^&&^ *p* < 0.01, ^&&&^ *p* < 0.001.

An ischemia‐reperfusion (I/R)‐induced AKI mouse model was used to further investigate the therapeutic potential of NP in reversing AKI in vivo.^[^ ^31^ ^]^ The experimental scheme is shown in **Figure** **6**A. Saline, tFNA, and NP were administered every 12 h after reperfusion. Mice were sacrificed on the third day post‐I/R surgery to collect the kidneys. HE and PAS staining were performed to more intuitively illustrate the histopathological damage in the kidneys (Figure 6B,D). Compared with the control group, the AKI group exhibited severe renal tubule damage with a disordered arrangement, characterized by massive coagulative necrotic areas, hyaline cast formation, loss of brush borders, and mucous exudate. Coagulated necrosis was reduced following the application of tFNA, with abundant hyaline casts and mucous exudates in the damaged renal tubules. Treatment with NP distinctly alleviated kidney tubular injury from I/R‐induced AKI, as evidenced by the remarkable decreases in tubular necrosis and hyaline casts, as well as the preservation of brush borders. To support our histopathological observations, semi‐quantitative analyses of the degree of tubular injury in the HE and PAS stained samples were employed (Figure 6C,E), in which the kidneys of each group were rated from 0 to 4 points according to the percentage of injured renal tubules.^[^ ^32^ ^]^ The results showed that the tubular necrosis score was highest in the AKI group and decreased following tFNA and NP treatment, with the most evident decline in the NP group.

Kidneys are rich in mitochondria due to their high energy demands for metabolism, nutrient reabsorption, and fluid and electrolyte balance.^[^ ^14a^ ^]^ Previous studies have shown that I/R injury often results in mitochondrial damage and dysfunction in the kidneys, which contributes to various pathological conditions of AKI and is responsible for its deleterious consequences.^[^ ^32^, ^33^ ^]^ To determine the protective effect of NP on mitochondria, TEM was used to observe mitochondrial morphology in renal tubular epithelial cells (RTECs). Representative sections are presented in Figure 6F. I/R injury led to extensive mitochondrial damage, characterized by swelling, the disorganization or loss of cristae, and fragmentation alongside matrix substance extravasation. The tFNA‐treated group showed minimal mitochondrial rupture but persistent swelling. However, after NP treatment the mitochondria were compact and elongated, with well‐organized cristae and normal morphology, suggesting that NP significantly reversed AKI‐induced mitochondrial damage in renal tubule cells. We also monitored the expression of CD68, the most reliable macrophage marker, using immunohistochemistry (Figure 6G,H). The results indicate that NP markedly relieved the AKI‐induced tubulointerstitial infiltration of inflammatory cells, including macrophages, which manifested as a remarkable decrease in CD68 expression. To assess the therapeutic efficacy of NP in ischemic AKI, we measured the expression of kidney injury molecule‐1 (KIM‐1), a key early biomarker of kidney injury, by immunohistochemistry (Figure 6I,J) and western blotting (Figure 6K; Figure S5A, Supporting Information). According to the immunohistochemistry results, I/R injury was associated with the distinct upregulation of KIM‐1 expression in the renal tubules, which was almost restored to the levels observed in the control mice after NP treatment. The western blotting results further supported this result; NP significantly reversed the elevation in KIM‐1 expression induced by ischemic injury. Overall, the I/R‐induced AKI mouse model was successfully established, and NP effectively treated the renal injury, as demonstrated by improvements in pathology, serology, microstructure, and protein expression.

### 2.8 NP Regulates I/R‐Induced Renal Tissue Apoptosis and Cell Cycle Arrest by Targeting p53

**Figure 7:**
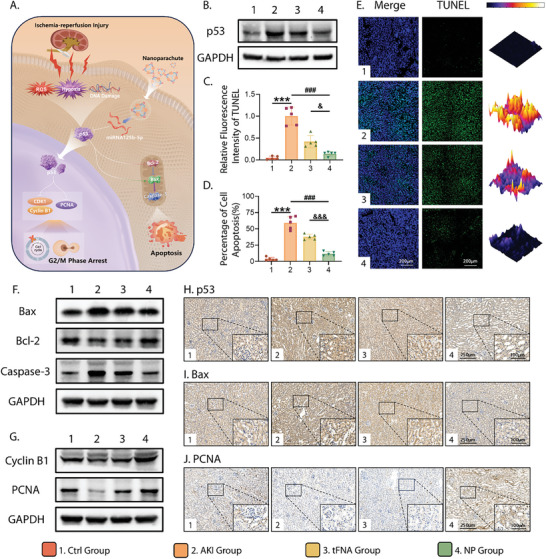
Regulation of I/R‐induced apoptosis and G2/M phase arrest of RTECs by NP via targeting of p53 protein. A) Schematic diagram of mechanisms involved in the protective effect of NP on I/R‐induced AKI. B) Protein expression of western blotting analysis of p53. C) Quantitative analysis of the relative TUNEL fluorescence intensity based on TUNEL staining. D) Quantitative analysis of the percentage of TUNEL‐positive cells among the total cells based on the TUNEL staining. E) Immunofluorescence images of kidneys with TUNEL staining (TUNEL: green; nucleus: blue; 3D thermal imaging: reconstruction of fluorescence intensity of TUNEL) (Scale bar: 200 µm). F) Protein expression of western blotting analysis of apoptosis‐related proteins: Bax, Bcl‐2, and Caspase‐3. G) Protein expression of western blotting analysis of cell cycle‐related proteins: Cyclin B1 and PCNA. H–J) Immunohistochemistry images of p53, Bax, and PCNA expression (Scale bars: 250 and 100 µm). All data were tested using one‐way ANOVA and *post hoc* analysis (Sidak. test) for multiple comparisons and presented as the mean ± standard deviation (SD) (*n* ≥ 3). The error bars represent the SD. Statistical analysis: (*) compared with the control group; *p < 0.05, **p < 0.01, ***p < 0.001 (^#^) compared with the AKI group; ^#^ *p* < 0.05, ^##^ *p* < 0.01, ^###^ *p* < 0.001 (^&^) compared with the tFNA group; ^&^ *p* < 0.05, ^&&^ *p* < 0.01, ^&&&^ *p* < 0.001.

P53, a nucleophosphorylated protein encoded by the well‐known tumor‐suppressor gene *p53*, has a molecular weight of 53 kD and comprises 393 amino acids. It is closely involved in cell cycle regulation, DNA repair, cell apoptosis, and other important biological functions.^[^ ^34^ ^]^ Previous findings support the crucial regulatory role of p53 in the pathogenesis of ischemia‐induced cell or tissue damage, such as myocardial infarction^[^ ^35^ ^]^ and I/R kidney injury.^[^ ^36^ ^]^ As illustrated in **Figure** **7**A and I/R injury can lead to cellular stress in RTECs, including ROS accumulation, hypoxia, and DNA damage, thereby activating p53 expression. Dysregulation of p53 is a pivotal contributor to both cell apoptosis and G2/M phase arrest through different signaling pathways, which are the underlying mechanisms of cell or tissue necrosis during AKI.^[^ ^37^ ^]^ On the one hand, upregulated p53 can give rise to mitochondrial damage and activate its subordinate apoptotic pathways, thereby inducing RTEC apoptosis.^[^ ^38^ ^]^ On the other hand, endonuclear p53 transcriptionally suppresses the binding of cyclin‐dependent kinase 1 (CDK1) to Cyclin B1 and the expression of proliferating cell nuclear antigen (PCNA), resulting in G2/M phase arrest in RTECs and the subsequent inhibition of cell proliferation.^[^ ^5^ ^]^ MiRNA‐125 has been found to interact with p53 and downregulate its expression, thereby coordinating the transcription of downstream apoptotic‐ and cell‐cycle‐associated proteins to suppress apoptosis and G2/M phase arrest.^[^ ^6^, ^39^ ^]^

Therefore, to investigate the mechanism underlying the NP‐induced amelioration of I/R‐induced AKI in mice, we investigated whether the nanocarrier NP could successfully and efficiently carry miRNA‐125 and regulate cell apoptosis and cycle pathways in the I/R environment. We first demonstrated that NP could normalize the upregulated p53 levels induced by AKI using western blotting (Figure 7B; Figure S5B, Supporting Information) and immunohistochemical staining (Figure 7H; Figure S5C, Supporting Information), indicating that p53 indeed served as the target of NP, whereas simple tFNA had marginal effects on p53 expression. TUNEL staining of the kidneys verified the effect of NP on cell apoptosis (Figure 7C–E). An AKI model significantly increased the number of apoptotic cells, manifested as a substantially enhanced green fluorescence intensity. Fluorescence decreased following intervention with tFNA and NP, with weaker intensity in the NP group, suggesting that NP was more conducive to reversing I/R‐induced apoptosis than tFNA. Consequently, we further investigated the expression levels of proteins relevant to the mitochondrial apoptotic pathway using western blotting. As demonstrated in Figure 7F and Figure S5D (Supporting Information), the levels of the pro‐apoptotic proteins Bax and Caspase‐3 were the highest in the AKI group and lowest in the NP group. However, the expression of the anti‐apoptotic protein Bcl‐2 showed the opposite trend, with the lowest level in the AKI group. A similar conclusion was observed after immunohistochemical staining for Bax (Figure 7I; Figure S5E, Supporting Information). These findings suggested that NP significantly enhanced the inhibitory effect of miRNA‐125 on I/R‐induced apoptosis via the activation of p53 to regulate the expression of the downstream proteins Bax, Bcl‐2, and Caspase‐3 in the mitochondrial apoptosis pathway.

Considering that p53 acts as a cross‐target between cell apoptosis and cell cycle arrest, we also monitored the alteration in cell cycle‐related protein expression. The expression levels of Cyclin B1 and PCNA in the kidney tissue of each group were measured by western blotting (Figure 7G; Figure S5F, Supporting Information). Compared to the control group, the protein levels of Cyclin B1 and PCNA were both significantly inhibited by I/R surgery, which matched the biological characteristics of I/R injury. TFNA administration restored the levels of these proteins to a limited extent; however, the enhanced amplitude was much lower than that after NP administration. The immunohistochemical staining of PCNA exhibited in Figure 7J and Figure S5G (Supporting Information) also pointed to a similar result; the brown staining symbolizing PCNA expression was distinctly light in the AKI group but recovered to an intensity similar to that in the control group after NP intervention. Collectively, the identification of the expression levels of cell cycle‐related proteins demonstrated that NP effectively eliminated the reduction in Cyclin B1 and PCNA expression caused by AKI, thereby inhibiting the G2/M phase arrest of cells, promoting cellular repair and proliferation, and ultimately accelerating damage repair.

### 2.9 Biocompatibility of NP

To evaluate the biocompatibility of the drugs, the mice were injected intraperitoneally with 100 µL of tFNA and NP every 6 h. Cardiac, liver, spleen, lung, and kidney samples were collected 48 h later, and HE staining was performed. Neither tFNA nor NP showed any pronounced toxicity to the internal organs (Figure S6, Supporting Information).

## 3 Conclusion 

To improve the application of RNA therapy in liver and kidney diseases, we introduced tFNA as a favorable nanocarrier for miRNA‐125 and optimized its 3D structure to successfully synthesize a novel nanocomplex (NP). miRNA‐125 was integrated into the framework of tFNA, which ensured that the nanoparticles retained their basic tetrahedral microshape and also provided a certain amount of protection from nucleic acid enzymes. In vitro studies have shown that NP exhibits excellent stability and sustained release. The application of a bioswitchable structure also provided an enzyme response function to promote the smooth opening of the nanoparticle and release of miRNA‐125 in the body, playing a significant anti‐injury role. In vivo tests further confirmed the ability of NP to passively target liver and kidney tissues, significantly extending its duration of action in the target organs. In ALF and AKI models, NP inhibited cell oxidative stress and G2/M phase arrest by regulating the expression of the Keap1/Nrf2 signaling pathway and p53 protein, respectively. It also blocked cellular apoptosis in the mitochondrial apoptosis pathway, achieving therapeutic effects in both ALF and AKI. In summary, we successfully developed a highly efficient, stable, and biocompatible miRNA delivery platform that supports the treatment of diseases associated with oxidative stress, cell cycle arrest, and apoptotic disorders, such as ALF and AKI. Considering the diverse and efficient biological roles of miRNAs, the clinical application of this miRNA delivery system could be further extended to elucidate the treatment and prevention of diseases in other systems.

## 4 Experimental Section

### Design, Fabrication, and Characterization of NP

All nucleic acid sequences required for NP preparation are listed in Table S1 (Supporting Information). Sangon (Sangon Biotechnology, China) synthesized these sequences. The nucleic acids were thoroughly mixed with TM buffer (50 mm MgCl_2_.6H_2_O and 10 mm Tris‐HCl), heated to 95 °C, and then rapidly cooled to 4 °C for at least 30 min. AGE (120 V, 25 min) and CGE (Bioptic Qsep 100, China) were used to analyze the assembled products. As previously reported, AFM and TEM were used to observe the morphology of NP.^[^ ^40^ ^]^ A Malvern Zetasizer (Malvern, UK) was used to analyze the hydrodynamic sizes and zeta potentials of the nanomaterials. To observe the change in the structure of NP after RNase H stimulation, the diluted RNase H was mixed with NP and incubated at 37 °C for 1 h; the products were analyzed by AGE and CGE.

### Metabolism and Distribution of NP In Vivo

The different fluorescently labeled nucleic acid sequences are shown in Table S1 (Supporting Information). Varioska LUX (Thermo Scientific, USA) was used to analyze the FRET results. First, 100 µL of fluorescently labeled NP was injected into the peritoneal cavity of 6‐week‐old Balb/c mice, and the mice were then sacrificed at different time points to harvest their internal organs, which were observed using an in vivo imaging system (IVIS Lumina, PerkinElmer).

### Advantages of NP as a miRNA Delivery Vehicle

The 5′ end of the miRNAs was labeled with Cy5 to prepare the drugs for administration to the miRNA, Entranster, and NP groups. Entranster was prepared according to the manufacturer's instructions (Engreen Biosystem, China). A 100 µL injection of miRNA drug (concentration 3000 nm) into the peritoneal cavities of the mice, and then analyzed them using an in vivo imaging system at different time points. The liver and kidneys were sliced and the cell nuclei were stained with DAPI, and observed the Cy5 fluorescence intensity using a laser confocal microscope. To obtain more specific results, a MicroRNAscope kit (Advanced Cell Diagnostics, USA) was employed, following the manufacturer's instructions. The stability of the nanomaterials was assessed using several tests. For the storage stability test, the nanomaterials were incubated at room temperature and 37 °C for 0–10 days. In the serum stability test, the nanomaterials were incubated at 37 °C in 2% and 10% serum for up to 24 h. All stability tests were performed using AGE.

### Establishment of Animal Models and Therapeutic Intervention

6‐ to 8‐week‐old Balb/C male mice were chosen for the animal experiment. All mice were obtained from Byrness Weil Biotech Ltd. (Chengdu, China). According to different disposal methods, the mice were allocated to the following four groups: Control (without APAP injection/I/R surgery), ALF/AKI (APAP injection/I/R surgery with no subsequent treatment), tFNA (APAP injection/I/R surgery with tFNA treatment), and NP (APAP injection/I/R surgery with NP treatment). An ALF model was established by the intraperitoneal injection of 350 mg kg^−1^ APAP (Pfizer, NY, USA) into Balb/C mice, with subsequent administration of saline, tFNA, or NP (100 µL) at intervals of 6 h. To induce AKI, the mice underwent I/R surgery. Briefly, after anesthetization, small incisions were made on the bilateral back skin of the mice, and the kidneys were then isolated and exposed. Non‐traumatic vascular clips were used to clamp the bilateral renal pedicles for 30 min. Reperfusion was initiated by the removal of the clips, and saline, tFNA, or NP was then administered intraperitoneally every 12 h after closing the incisions.

### Tissue Histology, IHC, and Immunofluorescence Staining

Fixed and paraffin‐embedded livers and kidneys were cut into 5 µm slices and then subjected to HE, PAS, IHC, and immunofluorescence staining, which were performed according to the product manual. Ten images of HE and PAS staining were captured under different visual fields to assess the impairment level of the liver and kidney tissues, with detailed scoring criteria as previously described.^[^ ^32^, ^41^ ^]^ The expression levels of Ly6G, CD68, Keap1, Nrf2, HO‐1, CD68, KIM‐1, p53, PCNA, Bax, Bcl‐2, and Caspase‐3 in the tissues were detected by IHC and immunofluorescence staining. All specimens were observed and images were captured via a slide scanner (3DHISTECH, Hungary) and a confocal laser microscope (FV3000; Olympus).

### DHE Staining

Freshly harvested kidney tissues were stored in liquid nitrogen for cryostat sectioning (thickness 5 µm). The tissue slices were incubated with 1 mm DHE solution for 30 min. Confocal images were captured using a confocal laser microscope (FV3000; Olympus).

### RT‐qPCR Array

Total RNA was isolated and purified using an RNA extraction kit (Thermo Fisher Scientific) following the manufacturer's protocols. The obtained RNA samples were reverse transcribed into cDNA, which was quantitatively amplified using a cDNA synthesis kit (Takara, Dalian, China). Target mRNAs were quantified using SYBR Green I PCR Master Mix in an RT‐qPCR thermal cycler (CFX96TM, Bio‐Rad). The primer sequences are listed in Table S2 (Supporting Information).

### Western Blotting Analysis

Total proteins from the liver and kidney tissues were extracted using lysis buffer. The extracted proteins were processed for western blotting as described previously.^[^ ^40^ ^]^ Antibodies against Keap1 (1:500, Abcam, Cambridge, UK), Nrf2 (1:200, Huabio, Hangzhou, China), HO‐1 (1:500, Huabio), KIM‐1 (1:1000, Abcam), p53 (1:300, Huabio), Cyclin B1 (1:200, Huabio), PCNA (1:500, Abcam), Bax (1:500, Huabio), Bcl‐2 (1:500, Huabio), Caspase‐3 (1:500, Huabio), and GAPDH (1:1000, Cell Signaling Technology, Boston, USA) were used as primary antibodies. Band intensities were visualized using an enhanced chemiluminescence detection system (Bio‐Rad).

### TUNEL Staining

Apoptosis in the liver and kidney sections was measured using a TUNEL staining kit (Roche, Mannheim, Germany). Apoptotic cells were labeled with fluorescein‐dUTP exhibiting green fluorescence, whereas DAPI‐stained nuclei emitted blue fluorescence.

### Statistical Analysis

Data were statistically evaluated using a one‐tailed Student's *t*‐test between two groups or a one‐way analysis of variance and post‐hoc analysis (Sidak. test) among three or more groups. GraphPad Prism software (GraphPad Software Inc., San Diego, CA, USA) was used for data analysis. Quantitative data were presented as the mean ± SD (*n* ≥ 3). Error bars in the graphs represent SD. A *p*‐value of <0.05 was considered to be statistically significant.

## Conflict of Interest

The authors declare no conflict of interest.

## Author Contributions

S.L. and Y.Z. contributed equally to this work. Y.L., X.C., and T.T. designed this study. S.L. and Y.Z. collected the data and wrote the manuscript. X.L., Y.C., T.Z., S.L., and Z.L. provided help during data collection and analysis.

## Ethics Approval Statement

All animal procedures were carried out in accordance with the protocols of the Animal Ethics Committee of Sichuan University (Ethics Number: WCHSIRB‐D‐2019‐100).

## Supporting information



Supporting Information

## Data Availability

The data that support the findings of this study are available from the corresponding author upon reasonable request.
