# Supplementary material for: Enzyme‐Responsive Nanoparachute for Targeted miRNA Delivery: A Protective Strategy Against Acute Liver and Kidney Injury
Source: Adv Sci (Weinh). 2024 Dec 24;12(10):2411210. doi: 10.1002/advs.202411210 (PMC11905073; doi:10.1002/advs.202411210)
Supplement: Supplementary file 1 — Supporting Information [file ADVS-12-2411210-s001.docx]

Supporting Information

Enzyme-responsive Nanoparachute for Targeted miRNA Delivery: A Protective Strategy Against Acute Liver and Kidney Injury

Songhang Li, ^#1^ Yuxuan Zhao, ^#1^ Xiaoying Lyu,^1^ Ye Chen, ^1^ Tao Zhang, ^1^ Shiyu Lin, ^2^ Zhiqiang Liu, ^1^ Xiaoxiao Cai,^1^ Taoran Tian, ^* 1^ and Yunfeng Lin^*1,3^

*^1^ State Key Laboratory of Oral Diseases, National Clinical Research Center for Oral Diseases, West China Hospital of Stomatology, Sichuan University, Chengdu 610041, P. R. China*

*^2^ Department of Oral Surgery, Shanghai Ninth People's Hospital, Shanghai Jiao Tong University School of Medicine; College of Stomatology, Shanghai Jiao Tong University, Shanghai 200011, P. R. China*

*^3^ College of Biomedical Engineering, Sichuan University, Chengdu 610041, P. R. China*

*^#^ Songhang Li and Yuxuan Zhao Contributed equally to this work.*

*^*^ Corresponding author: Taoran Tian and Yunfeng Lin*

Tel/Fax: 86-28-85503487

E-mail: yunfenglin@scu.edu.cn

State Key Laboratory of Oral Diseases, National Clinical Research Center for Oral Diseases, West China Hospital of Stomatology, Sichuan University, Chengdu 610041, P. R. China.

| Strand | Sequence (5'to 3') |
| --- | --- |
| Nanoparachute |  |
| S1 | ucacaaguAGGATGGGCATGCTCTTCCCGACGGTATTGGACCCTCGCATGAcucaggguTAAG |
| S2 | ucacaaguACATGCGAGGGTCCAATACCGACGATTACAGCTTGCTACACGAcucaggguTAAG |
| S3 | ucacaaguACGTGTAGCAAGCTGTAATCGACGGGAAGAGCATGCCCATCCAcucaggguTAAG |
| Cy5-miRNA125 | Cy5-ucccugagacccuaacuugugacuua |
| Matched-miRNA125 | Cy5-ucccugagacccuaacuugugacuua-BHQ2 |
| Mismatched-miRNA125 | Cy5-ucccugagacccuaacuugugagggg-BHQ2 |
| Cy3-miRNA125 | ucccugagacccuaacuugugacuua-Cy3 |
| FRET-miRNA125 | Cy5-ucccugagacccuaacuugugacuua-Cy3 |
| tFNA |  |
| tFNA-S1 | CAGTTGAGACGAACATTCCGATAAGTCTGAAATTTATCACGACCGCCATAGTAGACGTATCACCAGGTC |
| tFNA-S2 | GCTACACGATTCAGACTTATCGGAATGTTCGACATGCGAGGGTCCAATACCGACGATTACAGCTT |
| tFNA-S3 | GTGATAAAACGTGTAGCAAGCTGTAATCGACGGGAAGAGCATGCCCATCCACTACTATGGCGGTC |
| tFNA-S4 | CTCGCATGACTCAACTGGACCTGGTGATACGAGGATGGGCATGCTCTTCCCGACGGTATTGGACC |

**Table S1.** Sequences of DNA and RNA (capital letters represent DNA monomers, lowercase letters represent RNA monomers).


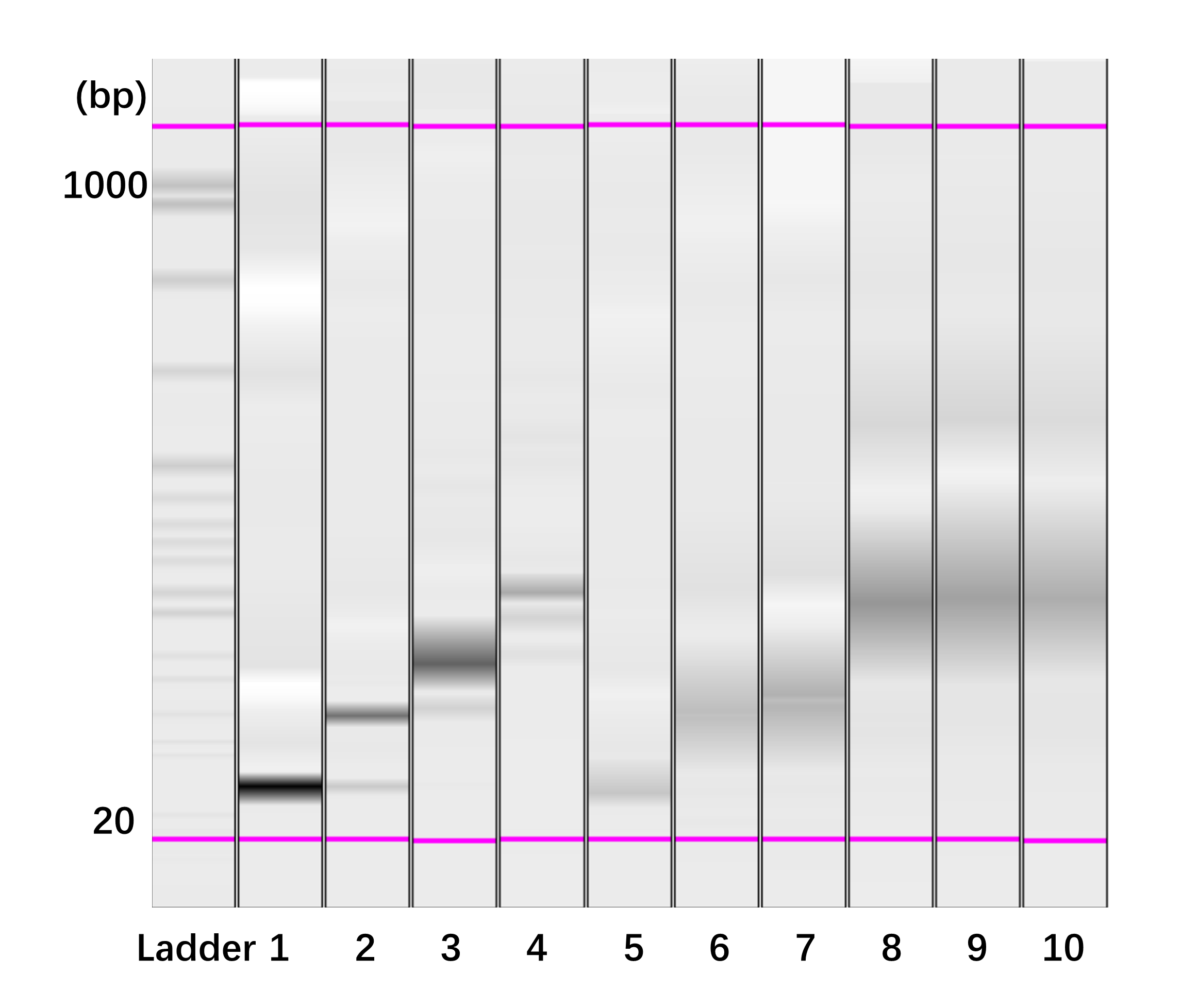


**Figure S1.** The successful synthesis of the tFNA and the NPs. Simulated Gel of CGE showed the successful synthesis of tFNA and the NPs (lane 1: tFNA-S1; lane 2: tFNA-S1+tFNA-S2; lane 3: tFNA-S1+tFNA-S2+tFNA-S3; lane 4: tFNA-S1+tFNA-S2+tFNA-S3+tFNA-S4; lane 5: S1; lane 6: S1+S2; lane 7: S1+S2+S3; lane 8: S1+S2+S3+Cy5-miRNA125; lane 9: S1+S2+S3+2×Cy5-miRNA125; lane 10: S1+S2+S3+3×Cy5-miRNA125).


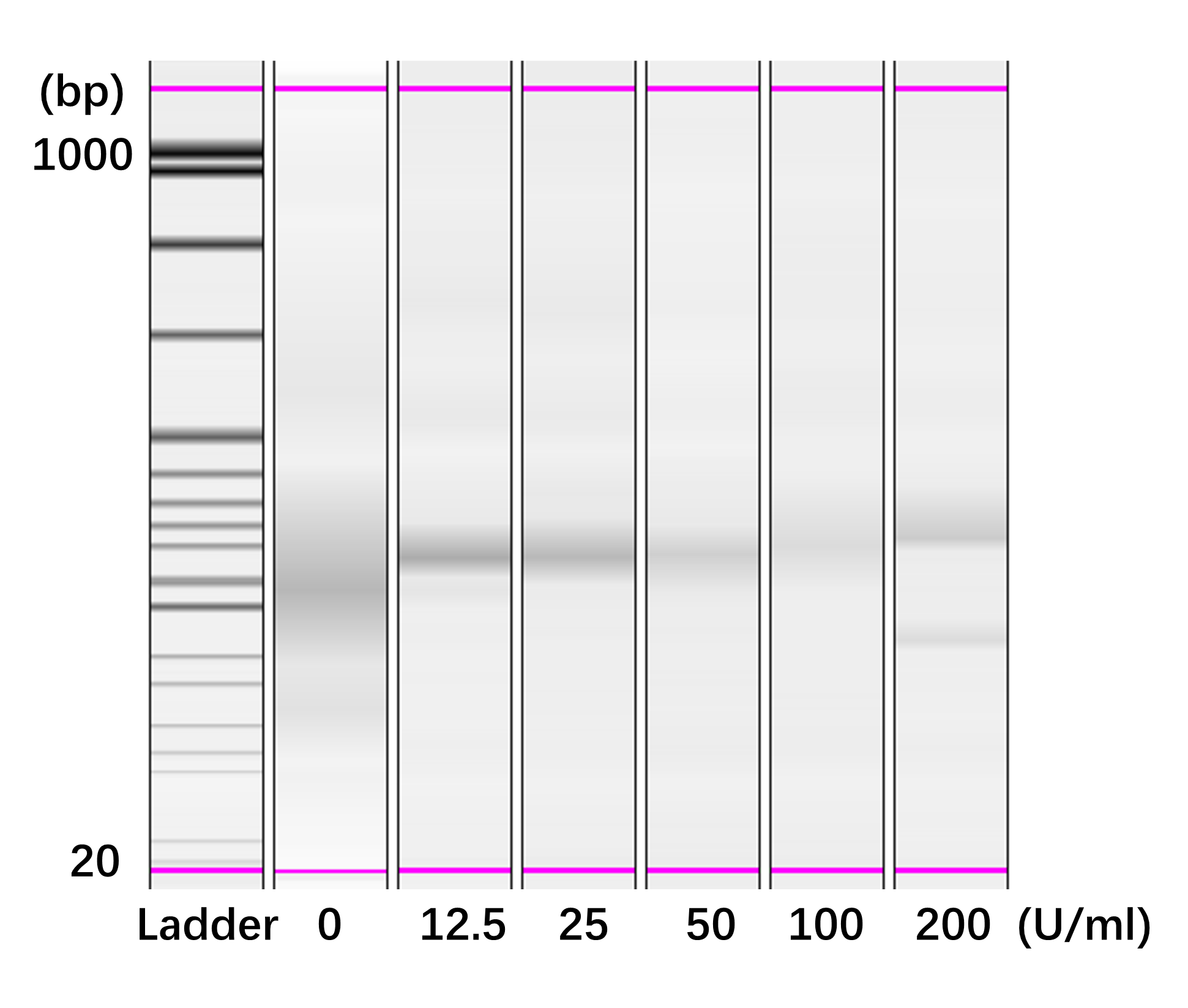


**Figure S2.** Simulated Gel of CGE showing the NPs undergo a conformational change in response to stimuli.
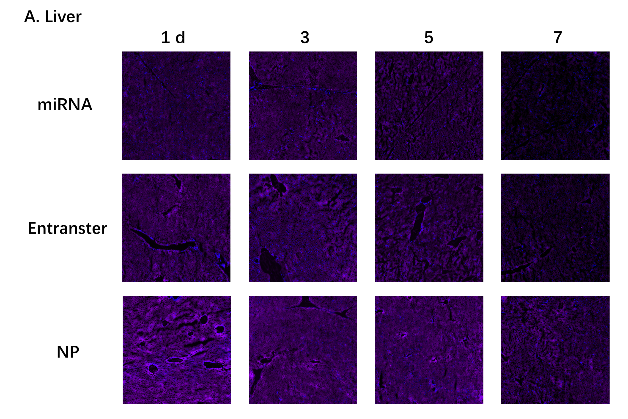

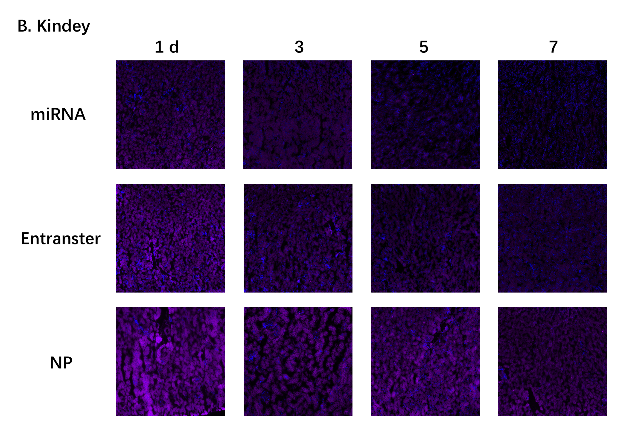


**Figure S3.** Fluorescence imaging of histological sections of livers (A) and kidneys (B) after injection of the miRNA, Entranster, and NP.


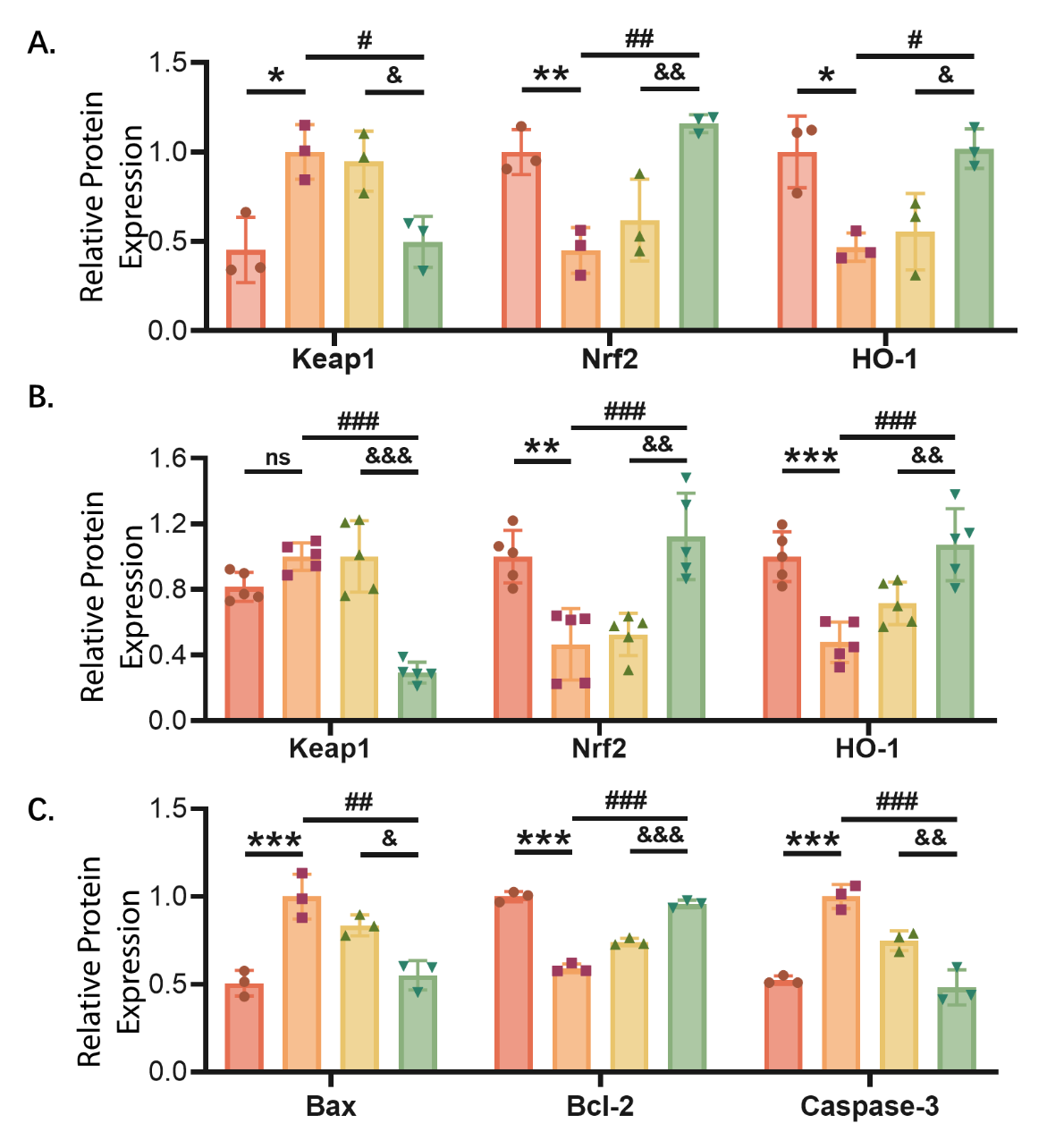


**Figure S4.** A) Quantitative analysis of Keap1, Nrf2, and HO-1 expression based on western blotting results. B) Quantitative analysis of Keap1, Nrf2, and HO-1 expression levels based on immunohistochemistry results. C) Quantitative analysis of apoptosis-related proteins expression based on western blotting results.


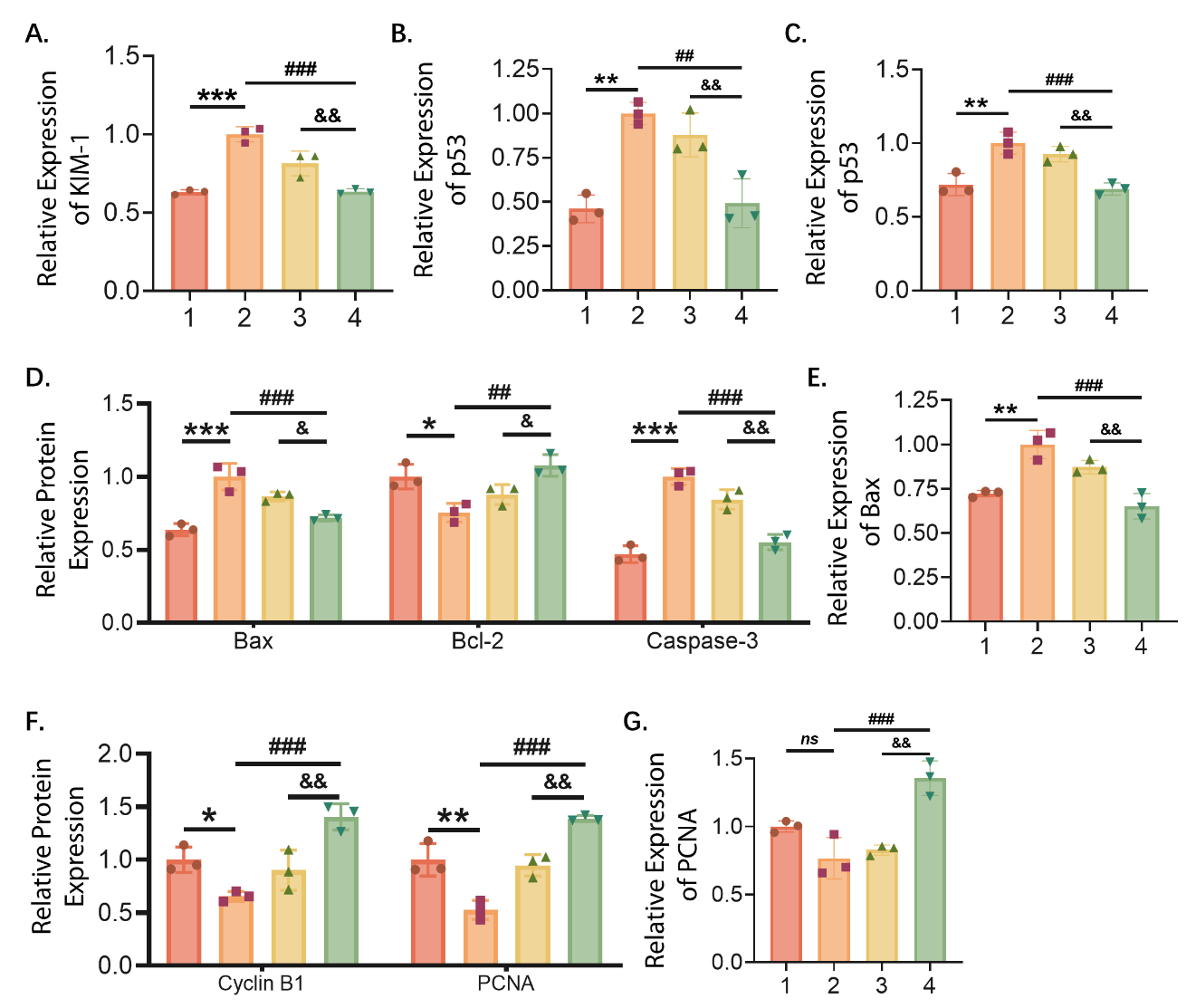


**Figure S5.** A) Quantitative analysis of KIM-1 expression based on western blotting results. B) Protein expression of western blotting analysis of p53. C) Quantitative analysis of p53 expression based on immunohistochemistry results. D) Quantitative analysis of apoptosis-related proteins expression based on western blotting results. E) Quantitative analysis of Bax expression based on immunohistochemistry results. F) Quantitative analysis of cell cycle-related proteins expression based on western blotting results. G) Quantitative analysis of PCNA expression based on immunohistochemistry results.

| cDNA | Sequence (5'to 3') |
| --- | --- |
| Keap1 | Forward GGAAAGTTTAAACGAGAAGCCTCTGGGCTCTG  Reverse GGAAATCTAGACCATCAGGATCTGCGTGTATT |
| Ugt1a6 | Forward AACTGCCAGAGCCTCCTGAA  Reverse TCAGCCAGGATCACACCACA |
| Gclc | Forward CTATCTGCCCAATTGTTATGGC  Reverse CCTCCCGTGTTCTATCATCTAC |
| Nqo1 | Forward GAAGACATCATTCAACTACGCC  Reverse GAGATGACTCGGAAGGATACTG |
| Gsta2 | Forward CTATGTTGAAGAGCTTGATGCC  Reverse ACTTGAAAACCTTCCTTGCTTC |

**Table S2.** Primer sequences of target mRNAs in RT-qPCR Array.

**
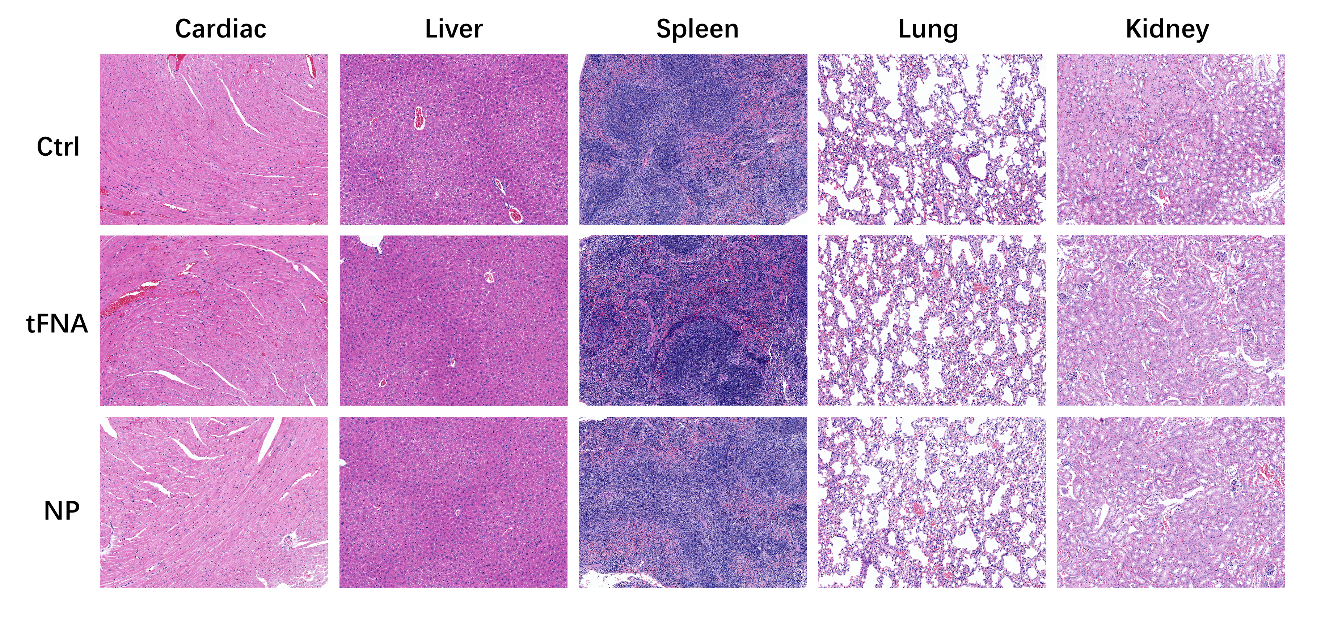
**

**Figure S6.** Biocompatibility of NP. HE staining of cardiac, liver, spleen, lung and kidney under different treatment to identify the non-toxicity of NP to vital organs *in vivo*.
